# The characteristics of a 12‐lead electrocardiogram measuring premature ventricular contractions originating from the tricuspid annulus using the clock position method

**DOI:** 10.1111/anec.13024

**Published:** 2022-12-27

**Authors:** Li‐juan Qu, Min Guo, Nan Zhang, Meng Sun, Rui Wang

**Affiliations:** ^1^ Department of Cardiology First hospital of Shanxi Medical University Taiyuan China

**Keywords:** electrocardiogram, premature ventricular beats, tricuspid annulus

## Abstract

**Objective:**

This study aimed to analyze the conventional surface electrocardiogram (ECG) characteristics of premature ventricular contractions (PVCs) originating from the tricuspid annulus and to investigate the efficacy of locating their origins according to ECG results.

**Methods:**

Eight patients who underwent radiofrequency ablation in the First Hospital of Shanxi Medical University (China) were included in the study. Pace mapping (PM) was used to analyze the characteristics of the PVCs originating from the tricuspid annulus recorded via 12‐lead body surface ECGs.

**Results:**

An R‐wave was found in leads I, V_5_, and V_6_. The QRS wave was narrower when the PVCs originated from the septum and shifted in lead V_3_ (R‐wave amplitude/S‐wave amplitude in the precordial lead—1). The QRS wave was broadest when the PVCs originated from the 7 to 9 o'clock position. The augmented vector left lead showed RS, QS, or RSR‐type waves with a low amplitude when the PVCs originated from the upper part of the annulus. When the PVCs originated from the lower part of the annulus, the augmented vector right lead reflected multidirectional and QS‐type waves.

**Conclusion:**

The ECG‐lead characteristics related to the origin of PVCs in the tricuspid annulus indicate some level of significance and can be used to formulate a specific diagnosis.

## Graphical abstract

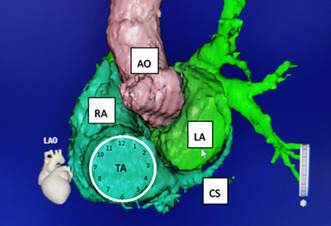

## 1 INTRODUCTION

The clinical manifestations of ventricular arrhythmias vary from being asymptomatic in mild cases to sudden cardiac death in severe instances by impacting the function and structure of the heart pumping cycle (Ge et al., 2017; Wen et al., 2015). The origins of ventricular arrhythmias are significantly correlated with age and sex (Tanaka et al., 2011). However, with respect to the nonoutflow tract ablation of premature ventricular contractions (PVCs), whether for children or adults, catheter ablation has demonstrated objective safety and effectiveness in successfully reducing or even eliminating the occurrence of arrhythmias (Gao et al., 2017). Idiopathic ventricular tachycardia/PVCs mainly originate from the right ventricular outflow tract (RVOT) and PVCs originating from the tricuspid annulus account for about 8% of those which originate from the nonoutflow tract. At the same time, the incidence of PVCs originating from the septum of the tricuspid annulus is about 2.8 times higher than it is for those originating from the free wall of the tricuspid annulus (Tada et al., 2007; Wang, Li, et al., 2019). Premature ventricular contractions originating from the tricuspid valve have attracted significant attention due to their specific anatomical position and adjacency to the Kent–His bundle (Tada et al., 2007). In this study, the pace mapping (PM) method was used to simulate PVCs originating from the tricuspid annulus. Through the observation and analysis of a 12‐lead electrocardiogram (ECG), this study provides evidence concerning the value of body surface ECG localization.

## 2 MATERIALS AND METHODS

### 2.1 Research subjects

A total of eight patients undergoing radiofrequency ablation in 2020 in the First Hospital of Shanxi Medical University (China) were selected for the study: They included two patients who had paroxysmal atrial fibrillation ablation, three patients who had right ventricular outflow catheter premature beat radiofrequency ablation, and three patients who underwent paroxysmal supraventricular tachycardia radiofrequency ablation. The study sample comprised four male and four female patients.

The inclusion criteria were as follows: (1) patients with normal development, a positive force body type, and a body mass index of 18.5–23.9 kg/m^2^; (2) patients of Han nationality, aged 30–60 years old; and (3) patients for whom corresponding indications for radiofrequency ablation were met.

The exclusion criteria were as follows: (1) patients with organic heart diseases, such as dilated cardiomyopathy, hypertrophic cardiomyopathy, valvular heart disease, and myocarditis, as confirmed by the relevant examinations; (2) patients with abnormal liver and kidney function (where “abnormal liver function” refers to chronic liver disease, such as liver cirrhosis, or significant biochemical disturbances, such as bilirubin levels higher than twice the upper normal limit and alanine transaminase/aspartate aminotransferase/alkaline phosphatase levels higher than three times the upper limit of the normal value, and “renal dysfunction” is defined as the need for chronic dialysis or a renal transplantation or a serum creatinine level ≥200 μmol/L); or (3) the presence of serious underlying clinical diseases, such as multisystem tumors. General information about the patients was collected. All study subjects provided signed informed consent for inclusion in the study, and the study protocol was approved by the hospital's ethics committee.

### 2.2 Research methods

Under local anesthesia, the right femoral vein was punctured, and a mapping electrode and catheter were inserted to perform the corresponding radiofrequency ablation.

**FIGURE 1:**
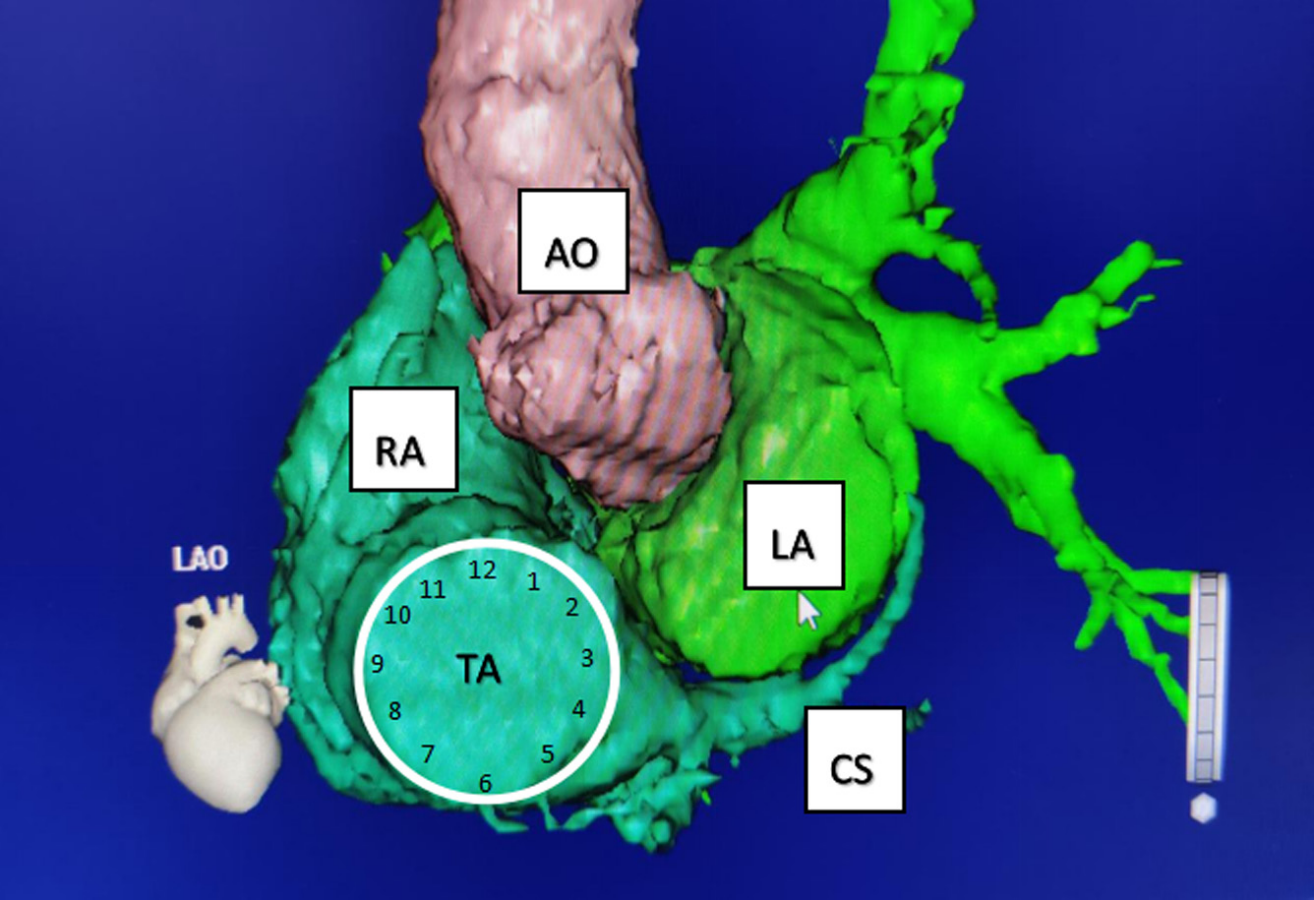
Adjacent relationship between the left anterior oblique 45° lower annulus was demonstrated by a three‐dimensional reconstruction (AO, aorta; CS, coronary sinus; LA, left atrium; RA, right atrium; TA, tricuspid annulus).

**FIGURE 2:**
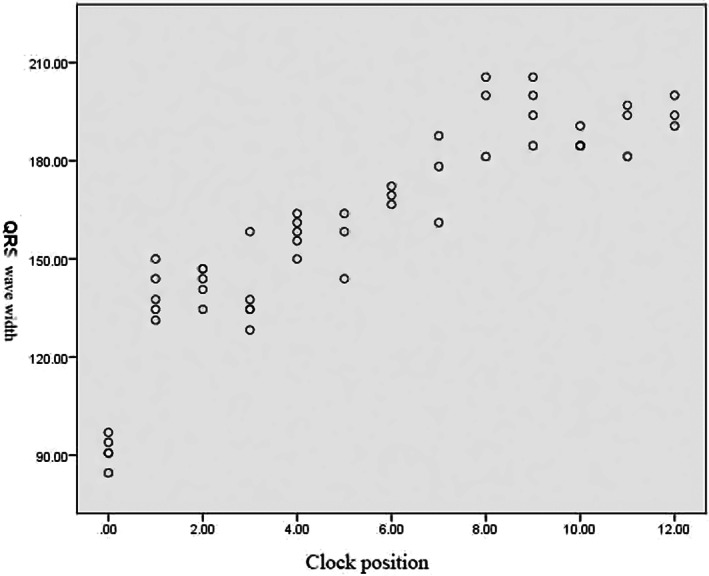
Scatter diagram of the QRS wave width and the corresponding clock position. In clockwise order, the width of the QRS duration gradually widens, with a maximum width at 7–9 o'clock, and then gradually decreases.

The tricuspid annulus and the RVOT were modeled under the guidance of an intracardiac ultrasound (Figure 1), and the catheter's proximity was monitored in real time. Marking the area with His bundle potential, the left anterior oblique was selected at a 45° angle, and the tricuspid annulus was evenly divided up (Figure 2) and marked out in a manner corresponding to a clockface. Thus, the vertex directly above the tricuspid valve ring was defined as point 12, and the ring was divided clockwise every 30°, corresponding with the points on a clockface. The tricuspid annulus was divided into the upper and lower parts and the free wall and septal sides, according to the lines that connected 3–9 o'clock and 12–6 o'clock, respectively. The free wall and septal sides were both further divided into three equal parts as follows: the anterior septum side (12–2 o'clock) and the anterior free wall side (10–12 o'clock); the middle septum side (2–4 o'clock) and the middle free wall side (8–10 o'clock); and the posterior septum side (6–4 o'clock) and the posterior free wall side (6–8 o'clock). The catheter can be used “anti‐C" method to ensure attach the top of the tricuspid annulus for PM. Pacing before the noticeable sense of shedding or a change in the direction of the force vector arrow is indicated in the three‐dimensional (3D) mapping system. Pace mapping was conducted synchronously on the ventricle side of the tricuspid annulus. The PM voltage was 7 V, the pulse width was 0.4 ms, and the PM circumference was 500 ms. Once the PM ECG was stabilized, electrocardiograms using 12 leads were measured and recorded in five groups at 1:10, after which their characteristics were analyzed.

### 2.3 Statistical analysis

The statistical analysis was carried out using the SPSS Statistics 23.0 software package. All data conformed to the homogeneity of variance and a normality test. Values were expressed as means ± standard deviations, and comparisons were made using an independent samples *t*‐test; *p*‐values < .05 were considered statistically significant. The enumeration data were expressed as a percentage (%), and a chi‐square (*χ* ^2^) test was conducted; *p*‐values below .05 were considered statistically significant.

## 3 RESULTS

### 3.1 Patient baseline information

There was no difference in age, sex, or comorbidities between the two groups (Table 1).

**TABLE 1:** Baseline patient data

	Male	Female	*p*
Age (year)	50.67 ± 6.21	45.98 ± 7.88	>.05
BMI (kg/m^2^)	22.72 ± 1.47	22.06 ± 2.52	>.05
Left ventricular ejection fraction (%)	56.85 ± 5.27	58.79 ± 5.63	>.05
Hypertension (number, %)	2 (50.00%)	2 (50.00%)	>.05
Diabetes(number, %)	1 (25.00%)	0 (0%)	>.05
Coronary heart disease (number, %)	2 (50.00%)	0 (0%)	>.05

Abbreviations: BMI, body mass index.

### 3.2 General features of the electrocardiogram

Each ECG lead linked to PVCs originating from the tricuspid annulus primarily indicated unidirectional waveforms, which were accompanied by a small number of multidirectional, low‐amplitude waveforms. With regard to the morphology of the left bundle branch block, the I, V_5_, and V_6_ leads primarily indicated unidirectional R‐waves.

The inferior wall lead changed depending on the clock position. The main wave direction of the inferior wall lead situated at 3 and 9 o'clock was inconsistent. Pace mapping was performed on the upper part of the annulus, and the dominant waves of leads II, III, and aVF were upward. The augmented vector left (aVL) lead had multidirectional waveforms, for example, low‐amplitude RS, QS, or RSR types, and when the pacing was at the lower part of the annulus, the main waves of the II, III, and aVF leads were downward. The augmented vector right (aVR) lead exhibited a multidirectional waveform. The leads of the anterior free wall of the annulus and the inferior wall of the anterior septum side essentially followed a positive direction. The main wave direction of the inferior wall leads in the middle of the annulus (II and III) was in opposition; the main wave of the lead III QRS wave was downward, and the main wave of the lead II QRS wave was upward, but the dominant waves of the II, III, and aVF leads were downward when the PVCs originated from the inferior wall. In all of the ECG time indicator positions, the amplitude of the R‐wave of lead I was larger than the sinus rhythm. The descending branch of the R‐wave may have indicated an incisure, and the ascending branch of the inferior wall lead may have reflected a similar result. The aVR lead indicated S‐ or RS‐type waves, while on the aVL lead, the QRS wave was basically positive, and the upper part of the annulus indicated rSR‐type waves.

**FIGURE 3:**
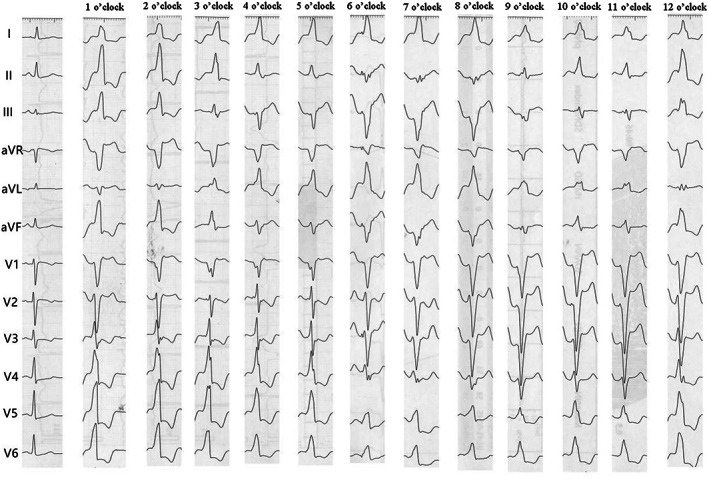
Tricuspid annulus pacing was performed according to the clock positioning method. In each group of images, there is a sinus rhythm on the left and a pacing diagram on the right.

The initial position of lead V_1_ for the PVCs originating at 2–6 o'clock showed a QS waveform. For PVCs originating at 7–12 o'clock, the QRS wave duration was significantly widened, and an R‐wave existed at the initiation of lead V_1_. The widest QRS wave (179.74 ± 15.56 ms, *p* < .05) occurred when the PVCs were located at 7–9 o'clock (Figure 3). After passing the 10 o'clock position, the QRS time limit dropped. In addition, for the chest, the V_2_ lead had the deepest S‐wave, the V_4_/V_5_ leads had the highest R‐wave, and the most frequent migration occurred in leads ≥V_3_, which was similar to its own migration lead.

### 3.3 Specific electrocardiogram features

Table 2 shows the ECG characteristics of each section. The QRS wave at points 1–2 was narrow, the aVL lead was dominated by the S‐wave, and leads V_2_–V_3_ appeared to indicate transitions. The R‐wave in lead III at 3 o'clock decreased significantly (2.36 ± 0.59) to only 1/3 of that in lead III at 2 o'clock. An incisure appeared in the descending branch of lead V_1_. From 4–6 o'clock, the amplitude of the R‐wave in lead I increased. The direction of the main wave in the inferior wall lead was reversed, and the chest lead shifted from V_2_–V_3_ to V_4_.

**TABLE 2:** Amplitude characteristics of ECG corresponding to pacing at 12 h.

	Norma		1 o'clock		2 o'clock		3 o'clock	
	R	S	R	S	R	S	R	S
I	4.72 ± 0.25	–	4.12 ± 0.29		4.36 ± 0.24		5.60 ± 1.06	
II	6.49 ± 0.30		11.99 ± 0.68		11.14 ± 0.24		7.76 ± 0.45	
III	2.36 ± 0.87		7.86 ± 1.22		6.60 ± 0.26		2.36 ± 0.59	2.55 ± 0.68
aVR		5.31 ± 0.23		7.86 ± 0.61		7.66 ± 0.20		6.88 ± 0.61
aVL	2.06 ± 0.68			2.26 ± 0.75	1.11 ± 0.24	1.05 ± 0.64	4.03 ± 1.45	
aVF	4.13 ± 0.32		9.83 ± 0.90		8.90 ± 0.24		4.71 ± 0.29	
V_1_	2.36 ± 0.40	8.55 ± 0.47		8.94 ± 1.11		8.02 ± 0.24		5.40 ± 0.16
V_2_	3.83 ± 0.52	9.73 ± 0.65	3.44 ± 0.74	12.38 ± 0.77	2.41 ± 0.38	12.09 ± 0.20	0.98 ± 1.70	6.49 ± 1.56
V_3_	4.42 ± 0.35	2.65 ± 0.56	7.47 ± 0.90	3.67 ± 0.63	5.72 ± 0.56	3.65 ± 0.44	5.21 ± 2.67	4.03 ± 1.33
V_4_	9.44 ± 0.23	1.45 ± 0.32	10.61 ± 0.77	–	9.56 ± 0.79	–	10.02 ± 2.06	–
V_5_	9.73 ± 0.85	–	12.38 ± 0.29	–	11.97 ± 0.49	–	10.52 ± 1.11	–
V_6_	7.96 ± 0.65	–	10.71 ± 0.94	–	10.49 ± 0.26	–	8.25 ± 0.29	–

	Norma		4 o'clock		5 o'clock		6 o'clock	
	R	S	R	S	R	S	R	S
I	4.72 ± 0.25	–	6.19 ± 0.72		7.57 ± 0.44		7.47 ± 0.84	
II	6.49 ± 0.30		5.60 ± 0.59		3.24 ± 0.49			4.42 ± 1.06
III	2.36 ± 0.87		1.25 ± 0.50	4.49 ± 1.39		7.27 ± 0.16		10.71 ± 0.34
aVR		5.31 ± 0.23		5.23 ± 0.44		5.01 ± 0.30		2.84 ± 0.90
aVL	2.06 ± 0.68		5.23 ± 0.97		7.07 ± 0.29		8.94 ± 0.45	
aVF	4.13 ± 0.32		3.24 ± 0.59		0.49 ± 0.84	4.12 ± 0.29		7.47 ± 0.74
V_1_	2.36 ± 0.40	8.55 ± 0.47		5.45 ± 0.16		7.18 ± 0.16		10.22 ± 0.16
V_2_	3.83 ± 0.52	9.73 ± 0.65	3.07 ± 1.87	6.19 ± 0.71	4.62 ± 1.54	7.66 ± 0.51	2.85 ± 0.44	11.89 ± 0.61
V_3_	4.42 ± 0.35	2.65 ± 0.56	6.19 ± 1.46	5.38 ± 0.50	4.52 ± 2.13	7.47 ± 0.74	1.57 ± 0.17	12.29 ± 1.03
V_4_	9.44 ± 0.23	1.45 ± 0.32	9.88 ± 1.30	–	8.16 ± 1.45	–	2.44 ± 0.46	3.43 ± 0.90
V_5_	9.73 ± 0.85	–	9.51 ± 0.37	–	9.04 ± 0.45	–	4.82 ± 0.84	–
V_6_	7.96 ± 0.65	–	7.08 ± 0.00	–	5.88 ± 1.82	–	5.30 ± 0.29	–

	Normal		7 o'clock		8 o'clock		9 o'clock	
	R	S	R	S	R	S	R	S
I	4.72 ± 0.25	–	7.17 ± 0.45		6.49 ± 0.23		4.42 ± 0.52	
II	6.49 ± 0.30		0.87 ± 0.28	1.24 ± 0.13		2.36 ± 0.12	4.13 ± 0.21	
III	2.36 ± 0.87			8.45 ± 0.34		9.44 ± 0.65		2.95 ± 0.26
aVR		5.31 ± 0.23		3.77 ± 0.16		2.36 ± 0.55		4.72 ± 0.63
aVL	2.06 ± 0.68		8.35 ± 0.45		7.96 ± 0.55		2.95 ± 0.46	
aVF	4.13 ± 0.32			4.72 ± 0.59		5.31 ± 0.23	1.47 ± 0.023	2.08 ± 0.63
V_1_	2.36 ± 0.40	8.55 ± 0.47		10.52 ± 1.95		12.39 ± 0.36		12.98 ± 0.46
V_2_	3.83 ± 0.52	9.73 ± 0.65	2.94 ± 1.34	13.17 ± 2.00	1.77 ± 0.11	14.55 ± 1.20	0.88 ± 0.20	19.17 ± 1.20
V_3_	4.42 ± 0.35	2.65 ± 0.56	2.06 ± 0.29	10.81 ± 1.03	0.88 ± 0.23	14.16 ± 0.95	0.12 ± 0.59	15.63 ± 0.65
V_4_	9.44 ± 0.23	1.45 ± 0.32	3.24 ± 0.29	–	5.31 ± 0.33		1.77 ± 0.12	6.19 ± 0.65
V_5_	9.73 ± 0.85	–	5.50 ± 0.61	–	4.72 ± 0.65		5.31 ± 0.23	–
V_6_	7.96 ± 0.65	–	4.81 ± 0.45	–	4.42 ± 0.66		6.19 ± 0.74	–

	Normal		10 o'clock		11 o'clock		12 o'clock	
	R	S	R	S	R	S	R	S
I	4.72 ± 0.25		4.13 ± 0.52		2.95 ± 0.12		5.01 ± 0.25	
II	6.49 ± 0.30		5.60 ± 0.12		4.72 ± 0.24		9.44 ± 0.71	
III	2.36 ± 0.87		1.47 ± 0.10	3.24 ± 0.22	2.06 ± 0.58	2.95 ± 0.44	5.60 ± 0.56	
aVR		5.31 ± 0.23		5.31 ± 0.25		4.13 ± 0.12		7.37 ± 0.45
aVL	2.06 ± 0.10		2.36 ± 0.26		1.77 ± 0.11			1.77 ± 0.86
aVF	4.13 ± 0.32		3.54 ± 0.40	1.77 ± 0.21	2.95 ± 0.23		7.67 ± 0.86	
V_1_	2.36 ± 0.40	8.55 ± 0.47		12.98 ± 0.42		12.39 ± 0.28	1.77 ± 0.78	10.91 ± 0.86
V_2_	3.83 ± 0.05	9.73 ± 0.65		19.46 ± 0.52		19.17 ± 0.14	3.83 ± 0.155	13.27 ± 1.20
V_3_	4.42 ± 0.35	2.65 ± 0.04		15.04 ± 0.32	0.59 ± 0.12	15.63 ± 0.32	5.90 ± 0.15	9.44 ± 0.98
V_4_	9.44 ± 0.23	1.45 ± 0.32	3.54 ± 0.17	4.42 ± 0.23	2.36 ± 0.17	7.96 ± 0.65	8.85 ± 0.17	–
V_5_	9.73 ± 0.85	–	6.49 ± 0.52	–	5.01 ± 0.58	–	9.44 ± 0.33	–
V_6_	7.96 ± 0.65	–	6.78 ± 0.25	–	6.19 ± 0.35	–	7.96 ± 0.74	–

## 4 DISCUSSION

The heart is conical and located in the lower mediastinum, with the bottom of the organ pointing to the upper and posterior sides. The tricuspid annulus is the largest heart valve and has a 3D structure within the organ, and its annulus can be separated from the diaphragmatic, anterior, and posterior lobes. The valve orifice measures approximately 7–9 cm^2^ and is inclined to the sagittal plane at a 45° angle. The joint site of the diaphragmatic and middle lobes is typically located at the opening of the coronary sinus to divide the right atrium and the right ventricle (Dahou et al., 2019; Hahn, 2016). The ventricular muscle forms a reversed structure at the tricuspid annulus indicating that part of the ventricular tissue can be located at the atrial level; however, the reversed structure is not obvious at the top (Wang, Long, et al., 2019). For PVCs originating from the tricuspid annulus, the ablation catheter can be bent into an inversed C shape. With the assistance of ultrasound, this ensures the stable adherence of the catheter and the effectiveness of the ablation (Luo et al., 2018; Wang, Li, et al., 2019). In this study, this method was used to carry out PM on the top of the tricuspid annulus to provide stable support for the catheter, thereby ensuring that the ablation catheter was attached to the pacing area. However, for the middle and lower part of the tricuspid annulus, the dragging method was used, which means pacing was carried out before the appearance of an obvious drop sensation or the 3D system showed that the force direction had changed. Accordingly, the ablation catheter was not inserted into the reflection area, but it was supplemented with an ultrasound catheter, which was positioned to ensure its stable adherence to the target area and stable pacing, thereby realizing a target area PM ECG.

A number of studies (Chen et al., 2018; Wu et al., 2013; Xu et al., 2018; Zhang et al., 2018, 2019) have shown that PVCs originating from the tricuspid annulus reflected extensive ECG characteristics; most I, aVL, V_5_, and V_6_ leads reflected unidirectional R‐waves, and the amplitude of the R‐wave in lead I was larger than the R‐wave in the sinus rhythm. However, the anterior lead may indicate different characteristics. The positive predictive value of PVCs derived from the tricuspid annulus interval was 96% when a QS‐wave appeared in the VI lead. Xuliang et al. (Chen et al., 2018) found an S‐wave in lead V_2_ < −1.81 mv as being the critical value for identifying PVCs originating from the interval or the free wall side of the tricuspid annulus. The tricuspid annulus can further be partitioned into three parts, that is, the former, middle, and latter areas. Furthermore, with changes in the origin sites of PVCs, a series of trend changes in the QRS wave will occur. The left anterior oblique at 45° is the conventional positioning for the coronary sinus electrodes. The tricuspid annulus can be divided into 12 equal parts, and the potential of the Kent–His bundle is typically positioned at 1 o'clock, while the coronoid sinus orifice is typically situated at 5 o'clock.

The mechanism of PVC is the premature excitation of the local myocardium, which leads to the depolarization of the whole ventricle. The tricuspid annulus is located at the front right side of the heart; therefore, the I, V_5_, and V_6_ leads show R‐waves. Meanwhile, PVC migration can be seen in leads ≥V_3_, and the width of the QRS wave further indicates the origins of the PVC. When PVCs originate from the ventricular septum, the time for left and right ventricles to complete activation is short, and the QRS wave is somewhat narrow. In addition, PVCs can originate between 7 and 9 o'clock, and these ones are the furthest removed from the normal conduction system of the heart. The right ventricle is locally activated first, and the left ventricle is activated thereafter, and the QRS wave time is wide. When PVCs originate from the upper part of the annulus, the aVL will include multidirectional waveforms, and when they originate from the lower part of the annulus, the aVR may indicate a multidirectional waveform. The upper part of tricuspid annulus is adjacent to the right ventricular outflow tract anatomically, and its PVCs show a similar performance in terms of ECG characteristics. Lu (Lu et al., 2016) reported that the ECG features of the tricuspid annulus and PVCs originating in the RVOT junction were as follows: The I, II, and III leads showed unidirectional R‐waves. The low level and polyphase of the QRS wave in the aVL lead showed a significant difference between the boundary–origin PVCs and TA/RVOT–origin PVCs. The aVL leads for PVCs that originated from the RVOT were primarily QS‐shaped. Wang (Wang, Long, et al., 2019) posited that the R‐wave of a lead I PVC originating from the top of the tricuspid annulus was larger than the sinus rhythm and that the aVL lead was mostly positive. However, the aVL leads of PVCs derived from the RVOT were mostly negative. The frontal lead indicated that the aVL lead pointed to the upper right quadrant, indicating the depolarization of the ECG vector from the center to the upper‐left side. The right ventricular outflow tract wounds from the front to the back of the heart and was adjacent to the posterior and superior tricuspid annulus. Therefore, it seems that the aVL leads in PVCs derived from the RVOT are purer in terms of morphology, and the bulk of waveforms present as single‐shaped. This study found that if PVCs were derived from the upper part of the tricuspid annulus, the aVL leads could reflect RS, QS, or RSR‐type waves with low amplitudes. This may have been due to the cancelation of left and right ECG vectors in conduction. For PVCs originating in the lower part of the annulus, this study found that the aVR lead could reflect multidirectional waveforms, with the bulk of wave morphologies being the QS type, and the aVL lead showed primarily a unidirectional R‐wave with no obvious notch.

The position of an ECG electrode is relatively fixed; however, for patients with different body types or underlying diseases, the position of the heart in the chest can vary. In this regard, positioning of the heart can be classified as normal, vertical, or transverse, or a clockwise/counterclockwise translocation of the heart. In the vertical position, the long axis of the heart is situated with a small angle to the sagittal plane. This angle is larger for a transverse heart position, which can be observed in obese patients or those with a short thorax. Changes in the position of the heart are not necessarily accompanied by pathological changes in the heart; however, an ECG may show corresponding changes. Yao et al. (Yao et al., 2001) found that the different positions of the heart had an obvious influence on potential changes in ECGs. According to this present study, for PVCs originating from the tricuspid annulus, ECG measurements can be predicted without a specific amplitude. When the electrode plate is placed in the standard position, variation in the characteristics of the QRS waveform and corresponding proportional relationships need to be analyzed to predict the corresponding location of the origin of the PVCs.

## 5 CONCLUSION

In conclusion, PVCs originating from the tricuspid annulus have specific ECG characteristics, and so the origin of PVCs can roughly be determined by ECG results. The findings of this study suggest that if the right‐side path has a pretransmission function and the component of the side path is increased (after stimulating the Kent–His bundle during the refractor period or giving the patient adenosine), its QRS waveform may be similar to the tricuspid annulus source's chamber velocity source. Familiarity with the ECG characteristics of PVCs originating from the tricuspid annulus may therefore provide a useful reference for the positioning of a bypass tract.

## AUTHOR CONTRIBUTIONS

Rui Wang and Nan Zhang were involved in the conception and design of the research. Rui Wang, Nan Zhang, and Lijuan Qu were involved in the acquisition of data. Lijuan Qu and Rui Wang were involved in the analysis and interpretation of the data. Lijuan Qu was involved in statistical analysis and wrote the manuscript. Rui Wang, Min Guo, and Meng sun were involved in obtaining financing and critical revision of the manuscript for intellectual content. All authors read and approved the final draft.

## FUNDING INFORMATION

National Natural Science Foundation of China (No.82000426); the Natural Science Foundation of Shanxi Province (No:201801D121222 and 201801D121337 to Min Guo); PhD Fund of The First Hospital of Shanxi Medical University (No.YB161702).

## CONFLICT OF INTEREST

All authors have contributed significantly to the manuscript and declare that the work is original and has not been submitted or published elsewhere. None of the authors have any financial disclosure or conflict of interest.

## DATA AVAILBILITY STATEMENT

The datasets used and/or analyzed during this study available from the corresponding author upon reasonable request.

## ETHICAL APPROVAL

This study was conducted in accordance with the Declaration of Helsinki. This study was conducted with approval from the Ethics Committee of First Hospital of Shanxi Medical University.

## INFORMED CONSENT

Written informed consent was obtained from all participants.
